# Experiences of a School Medical Officer

**Published:** 1909-08-07

**Authors:** 


### Public Health and Hygiene.

### EXPERIENCES OF A SCHOOL MEDICAL OFFICER.

(From a Correspondent.)

In the last twelve months a new sphere in school work has been found for the junior ranks of the profession. At present provision is made only for "inspection" of children; but the inevitable and only reasonable sequel is some form of treatment which will mean the employment of still more men in this service. In all parts of the country experience on this matter is identical. Parents practically ignore the recommendations of the medical officers. Indeed, if from those who do receive attention those obtaining it in hospital were deducted, the remainder would probably be a decimal point per cent. Compulsion would be impossible, and, indeed, since the introduction of the Education Act, "must" has always meant "rates" in the end. We may conclude, therefore, that treatment at the expense of the rates, and, possibly, the national exchequer, is inevitable. Men meditating entering on this work, then, may be glad to hear how those first in the field have fared.

Though it is frequently said that this work is for recent graduates the remark is not strictly true, as some special training after qualification is a *sine qua non*. A man must have held a residency in a children's hospital or isolation hospital, or both; he must have had experience in at least one of the specialities—eyes, nose, and throat, etc.; and now it is becoming more and more evident that the possession of the D.P.H. is almost essential. School work is really a branch of preventive medicine, and it cannot be divorced from it. It should be controlled from the medical officer of health's office, and this is the view the Board of Education now takes. Bearing this in mind, it is obvious that two or three years at least must pass before the newly qualified man can reasonably hope to get a good school appointment. But a good school appointment is a small thing compared with a decent private practice, so that the man who has capital might be comfortably settled by the time he is prepared for obtaining school work.

Financially, the possession of a school post does not mean much. The average salary is £250, so that the house surgeon of a good hospital is nearly as well off, and even the junior posts in asylums, keeping in mind the fact that in them board and lodgings are provided, are equally good. The prospects for the future are vague. Many of us hope that it will pave the way to public health appointments; and others think, with what grounds it is hard to say, that the status of the school officer will greatly improve. Certain advantages of the work are obvious. Saturday afternoons and evenings and Sundays are the man's own, and he does not fear the bell or telephone at night. Moreover he has a regular salary.

What of the work itself? My work lies in a large county with several urban districts and one or two large, thinly populated rural areas. A prime difficulty is that of transit. For that one has to depend on trains and a bicycle, and these have a great influence on one's enjoyment or the reverse. Some men use motor bicycles: presumably they are highly insured. Another difficulty of the county officer is that of feeding. He is for the most part dependent on country inns for his mid-day meal. In one inn I could get nothing but bread and cheese and beer, and the bread was straight from the oven. In another, of very similiar stamp and circumstance, I lunched off roast pheasant at absurdly small cost.

Of these ups and downs the town officer can know nothing, and I for one would not change with him. The ride to school on a spring morning requites one for winter discomforts many times over, and it keeps one very "fit."

In the school itself there is much monotony about the work, unfortunately. One is, after all, inspecting fairly normal individuals, so that there is little to record save the occurrence of verminous conditions, decayed teeth, enlarged tonsils and adenoids, and cases of refractive errors. This, of course, is the fault of the scheme. In every school there are unhealthy children, and if one were permitted to devote oneself to a "selective" inspection the work would be much more interesting. As things are, the great aim seems to be to secure a statistical record of heights and weights, etc., of great masses of children—very interesting, no doubt, for the anthropometrist, but neither highly practical nor utilitarian. When "entrants" and "leavers" are being examined in routine fashion there may be so many of these to attend to that no time is available for those with obvious defects at other ages.

After some practice twelve, or even fifteen, children per hour can be inspected, and this rate of work must be maintained if the large numbers of children due for inspection are to be overtaken. Possibly fifty children are inspected daily.

Auscultation in a consulting room is one thing; in a room containing twenty children with iron-shod, restless feet it is quite another. Listening for mitral murmurs amidst such distracting noises, a stuffy room, fractious children, and an obstructive or garrulous teacher, is not of the easiest or good for the temper. When this process goes on day by day, week in and week out, and the prospect of the years is contemplated, summation of stimuli wins, and the inspector seeks pastures new. We have here struck a real difficulty, and one that will be more in evidence in the next few months. Already some authorities have had more than one medical officer, and, undoubtedly, the monotony of the work is the chief cause of the change. For this reason alone it is advisable that the work be done by general practitioners, under the supervision of a small permanent staff. The amount of work each man would have to do would be too small to produce ennui, and yet be sufficiently remunerative to induce the junior practitioner to compete for the post. In addition this policy would tend to allay the fears of medical practitioners that by degrees all their work will be taken out of their hands.

But the work is not without interest. When figures are compiled and one moves from district to district, interesting comparisons become possible. The frequency of measles and whooping cough in early life, of enlarged tonsils and decayed teeth, the relative frequency of spinal curvature, surprise one; still more, perhaps, the extreme commonness of deformed chests, and this seems to develop after the fifth or sixth year, strikes one at once. Unsuspected conditions are found in the way of heart lesions and spinal curvatures, also some remarkably interesting cases that have never found their way into consulting room or clinique.

Again, " mental deficients " afford interest to the keen clinician. The enormous number of these cases and the enormous need for something to be done for their control is a very real problem, and one that will have to be grappled with on a national scale at an early date. When one pauses to consider their ætiology, their dependence on others, and their present liberty to propagate their likes, one realises the influence they have on national physique, economics, and the national future.

The tuberculosis problem is another that the school medical officer has to help to solve. Whether phthisis is a common disease of childhood or not is now a subject of debate; but other tuberculous lesions are extremely frequent, and an adequate answer to the question of what is to be done for them has still to be found. There are all sorts of questions similarly awaiting answer. "Free meals," " Open-air schools," " Special schools," " Exceptional cases," are frequent phrases in the vocabulary of the school medical officer. Only a few weeks at the work serve to make him a social reformer and the problems of his work are the salt of life to him. If he were relieved of the monotony of routine inspections his life would be a real joy.

